# Sialic acid level reflects the disturbances of glycosylation and acute-phase reaction in rheumatic diseases

**DOI:** 10.1007/s00296-013-2921-y

**Published:** 2013-12-18

**Authors:** Lech Chrostek, Bogdan Cylwik, Ewa Gindzienska-Sieskiewicz, Ewa Gruszewska, Maciej Szmitkowski, Stanislaw Sierakowski

**Affiliations:** 1 Department of Biochemical Diagnostics, Medical University, Waszyngtona 15A, 15-269 Bialystok, Poland; 2 Department of Rheumatology and Internal Diseases, Medical University, Bialystok, Poland

**Keywords:** Sialic acid, Rheumatic diseases, Glycosylation abnormalities

## Abstract

In the rheumatic diseases, the changes in the carbohydrate part of serum glycoproteins occur and these abnormalities can be monitored by serum level of total and free sialic acid. The aim of this study was to evaluate the total and free sialic acid level as a marker of inflammation activity (TSA) and the changes in glycosylation of blood glycoproteins (FSA) in rheumatoid arthritis (RA), systemic sclerosis (SSc) and systemic lupus erythematosus (SLE). Studies were carried out in 50 patients with RA, 24 with SLE and 32 with SSc. TSA concentration was measured with an enzymatic, colorimetric method and FSA with a thiobarbituric method. The serum levels of TSA in RA and SLE patients were significantly increased compared to controls and in RA patients were higher than that in SSc patients. The mean serum level of FSA in RA patients was significantly higher, but in SSc patients significantly lower than that in the controls, and in RA patients was significantly higher than in SLE and in SSc patients. All acute-phase proteins were changed: Positive acute-phase proteins were elevated, and the negative protein was decreased. The positive acute-phase proteins positively correlated with the levels of TSA and FSA in RA and SSc patients. In SLE patients, TSA positively correlated with haptoglobin and α1-antitrypsin. In RA patients, there was the positive correlation of TSA and FSA with DAS 28. The changes in the serum levels of TSA and FSA in the course of rheumatic diseases could reflect the abnormalities in glycosylation/sialylation patterns of glycoproteins induced by acute-phase response.

## Introduction

There are evidences that the changes in the protein glycosylation during the course of rheumatic diseases are implicated in the pathogenesis of rheumatic diseases [1]. The association between changes in the glycosylation homeostasis and pathogenic mechanism in rheumatic diseases is so constant and characteristic that it has been referred as “sugar printing the rheumatic diseases” [2]. More recent works indicate that not only the lack of galactose residues on immunoglobulin G itself but, rather, the concomitant absence of terminal sialic acid may be responsible for pro- and anti-inflammatory activity of IgG [3, 4]. It has been documented that the lack of galactose residues results in a concomitant lack of terminal sialic acid residues and increases the affinity for activating Fcγ receptors [5, 6]. Otherwise, the presence of glycan structure terminating in sialic acid residues reduces affinity of antibodies to cellular Fc receptors and enhances the anti-inflammatory activity exerted by G0 glycoforms of IgG [3, 4].

In the pathogenesis of rheumatic diseases are also implicated the altered glycosylation of other than IgG plasma proteins such as fibronectin, transferrin, α1-acid glycoprotein and haptoglobin [7–9]. Structurally, these are glycoproteins terminated with sialic acid residues, and their levels in the blood may be non-specific markers of the disease activity [10]. Therefore, the total level of sialic acid (TSA) can measure the activity of acute-phase reaction, but the free sialic acid (FSA) can measure the disturbances in sialylation of these glycoproteins during disease [11, 12]. The aim of this study was to evaluate the total and free sialic acid level as a marker of inflammation activity and the changes in glycosylation of serum glycoproteins in the course of rheumatoid arthritis (RA), systemic sclerosis (SSc) and systemic lupus erythematosus (SLE).

## Methods

### Patients

Studies were carried out in 50 patients with RA (40 females and 10 males) aged from 25 to 85 years (mean age 55 years), 24 with SLE (22 males and 2 females) aged from 23 to 70 years (mean age 40 years) and 32 with SSc (27 females and 5 males; 18 with diffuse cutaneous SSc and 14 with limited cutaneous SSc) aged from 19 to 71 years (mean age 47 years) admitted to the Department of Rheumatology and Internal Diseases. The duration of the RA was from 2 months to 25 years, SLE from 1 to 21 years and SSc from 3 months to 15 years. The diagnosis of the disease was made on the basis of criteria given by American College of Rheumatology. RA patients were taking non-steroidal anti-inflammatory and disease-modifying antirheumatic drugs such as methotrexate and sulfasalazine. The RA activity was evaluated by disease activity score (DAS 28) according to the following formula which included the number of tender (t28) and swollen (s28) joints, ESR and VAS:

$${\text{DAS 28}} = 0. 5 6\times {\text{sqrt}}\left( {\text{t28}} \right) + 0. 2 8\times {\text{sqrt}}\left( {\text{s28}} \right) + 0. 7\times { \ln }\left( {\text{ESR}} \right) + 0.0 1 4\times {\text{VAS}}$$

To assess disease activity in SLE patients, the Physician’s global assessment (PGA) was used. It based on a visual analog scale 0–100 mm, in which score “0” indicates an inactive disease (none of patients), score “1”—mild disease (3 patients), score “2”—moderate (13 patients) and score “3”—severe disease (8 patients). The 42 control subjects (33 women and 9 men) were recruited from mixed healthy hospital workers (mean age 42 years, range 26–65). Blood samples were collected from each patient once on admission from a peripheral vein after a 12-h fasting. Patients and controls gave informed consent and participated in the study. The laboratory characteristics of the healthy subjects and patients are shown in the Table 1.

**Table 1:** The results of laboratory tests in patients and controls

	ESR (mm/h)	Hb (g/L)	PLT (10^3^/μL)	RF (IU/mL)	IgG (g/L)	Anti-CCP (U/mL)
Controls (*n* = 42)	6.0 1.0–9.0	13.7 11.3–15.5	243 144–386	21.0 20–25	11.4 7.3–13.3	0,5 0.5–1.3
RA (*n* = 50)	40.0 6.0–100 *P* < 0.001	11.9 7.9–15.2 *P* < 0.001	290 148–490 *P* = 0.021	71.0 20–898 *P* < 0.001	11.4 7.0–18.8 *P* = 0.903	41.3 0.5–1,373 *P* < 0.001
SLE (*n* = 24)	32.5 10.0–105.0 *P* < 0.001	12.2 8.8–13.6 *P* < 0.001	242 106–588 *P* = 0.903	20.0 20–27 *P* = 0.007	9.8 6.3–16.8 *P* = 0.062	0.5 0.5–26.9 *P* = 0.511
SSc (*n* = 32)	20.0 4.0–70.0 *P* < 0.001	11.9 8.1–14.8 *P* < 0.001	253 107–561 *P* = 0.454	20.0 10.6–96.8 *P* < 0.001	14.1 8.8–23.2 *P* = 0.008	0.5 0.5–47.9 *P* = 0.306

Data are medians and ranges. The differences between patients and controls estimated by Mann–Whitney *U* test. *RA* rheumatoid arthritis, *SLE* systemic lupus erythematosus, *SSc* systemic sclerosis, *P* < 0.05 significant difference, *ESR* erythrocyte sedimentation rate, *Hb* hemoglobin, *PLT* platelets count, *RF* rheumatoid factor, *IgG* immunoglobulin G, *Anti-CCP* autoantibodies to cyclic citrullinated peptide

### Laboratory analysis

ESR (normal range <10 mm/h for men and ≤20 mm/h for women up to 50 years, and ≤30 mm/h for women more than 50 years) was determined by Westergren method on the Sediplus S 2000 (Sarstedt, Germany). Hemoglobin level (normal range for women 12–16 g/dL and for men 14–18 g/dL) and PLT (normal range 130–350 × 10^3^/μL for adults) were determined by using routine methods on the hematological analyzer Advia 120 (Bayer, USA). The transferrin (TRF) concentration (normal range 2.0–3.6 g/L) was determined by the immunoturbidimetric method using Siemens test (Siemens Healthcare Diagnostics, Marburg, Germany) on BN II analyzer. CRP (*CRP Vario* test: normal range ≤5 mg/L), RF (*Quantia RF* test; lower than 30 IU/mL are considered normal, and results between 30 and 50 IU/mL as weakly positive), immunoglobulin G (*Immunoglobulin G* test; normal range 5.52–16.31 g/L for female; 5.40–18.22 for male), α1-antitrypsin (AAT) (*Quantia A1*-*Antitrypsin* test; normal range 0.9–2.0 g/L), α1-acid glycoprotein (AGP) (*Quantia A*-*1*-*AGP* test; normal range 0.5–1.2 g/L) and haptoglobin (*Haptoglobin* test; normal range 0.35–2.50 g/L for female; 0.14–2.58 g/L for male) were measured with an immunoturbidimetric methods (Abbott, Wiesbaden, Germany) on the Architect c8000 analyzer (Abbott Laboratories, Abbott Park, USA). Autoantibodies against cyclic citrullinated peptide (anti-CCP) were determined with a chemiluminescent microparticle immunoassay (CMIA) using the *anti*-*CCP* test (Abbott, Wiesbaden, Germany) on an Architect i2000 analyzer (Abbott Laboratories, Abbott Park, IL, USA). A cutoff of 5.0 U/mL was chosen, whereby a result of ≥5.0 was considered positive and a result of <5.0 was considered negative. 14 of RA, 2 of SSc and 2 of SLE patients were RF positive (RF+).

TSA concentration was measured with a colorimetric, enzymatic method, using *Enzy*-*Chrom Sialic Acid Assay Kit* (BioAssay Systems, Hayward, CA, USA) on the Microplate Fluorescence Reader FL600 (Bio-Tek, USA).

FSA was determined using the thiobarbituric method of *Skoza and Mohos* [13]. All reagents were from Sigma-Aldrich Chemie GmbH. Measurements were performed at 549 nm on the spectrophotometer Shimadzu UV-1202 (Shimadzu Europa GmbH, Duisburg, Germany).

### Statistical analysis

The differences between the tested and control groups were evaluated by Mann–Whitney *U* test and the correlation between SA and acute-phase proteins by the calculation of Spearman^’^s rank correlation coefficient. The effect of the activity of rheumatic diseases on the TSA and FSA values was evaluated by the ANOVA rank Kruskal–Wallis test. Because the chance of finding one or more significant differences in three tested groups was only 14.26 % (Bonferroni correction), the post hoc multiple comparison analysis was performed. The GraphROC for Statistica version 10 was used to evaluate the diagnostic sensitivity, specificity, positive (PPV) and negative predictive values (NPV), and the area under the receiver operating characteristic (ROC) curve.

## Results

Table 1 presents the results of laboratory tests in the blood of patients and the comparison of them with the results of the controls. The mean values of ESR and RF were significantly higher but Hb significantly lower, in patients with rheumatic diseases than that in the controls. The levels of PLT and anti-CCP were higher only in RA patients but IgG in SSc patients. The serum levels of acute-phase proteins are shown in Table 2. All acute-phase proteins in patients with rheumatic diseases were significantly changed in comparison with the controls: positive acute-phase proteins (CRP, AGP, HP and AAT) were elevated and the negative (TRF)—decreased. Total protein level was significantly diminished in RA and SLE patients.

**Table 2:** The results of acute-phase proteins in patients and controls

	CRP (mg/L)	AGP (g/L)	HP (g/L)	AAT (g/L)	TRF (g/L)	TP (g/L)
Controls (*n* = 42)	0.75 0.3–3.2	0.73 0.45–1.25	0.98 0.60–2.26	1.26 1.17–1.57	2.44 1.01–3.50	7.1 6.4–7.9
RA (*n* = 50)	11.90 0.80–106 *P* < 0.001	1.07 0.53–2.54 *P* < 0.001	2.12 0.76–3.57 *P* < 0.001	1.60 1.04–2.82 *P* < 0.001	2.03 1.29–3.15 *P* = 0.004	6.7 4.7–10.5 *P* = 0.010
SLE (*n* = 24)	5.0 0.7–57.1 *P* < 0.001	1.06 0.76–2.30 *P* < 0.001	1.62 0.85–3.0 *P* < 0.001	1.45 1.20–2.28 *P* < 0.001	1.91 1.48–3.76 *P* = 0.003	6.1 5.1–7.4 *P* < 0.001
SSc (*n* = 32)	2.6 0.5–66.5 *P* < 0.001	1.15 0.51–2.81 *P* < 0.001	1.56 0.60–3.57 *P* < 0.001	1.56 1.11–2.43 *P* < 0.001	2.09 1.55–2.70 *P* = 0.003	7.0 5.8–8.4 *P* = 0.144

Data are medians and ranges. The differences between patients and controls estimated by Mann–Whitney *U* test. *RA* rheumatoid arthritis, *SLE* systemic lupus erythematosus, *SSc* systemic sclerosis, *P* < 0.05 significant difference, *CRP* C-reactive protein, *AGP* α1-acid glycoprotein, *HP* haptoglobin, *AAT* α1-antitrypsin, *TRF* transferrin, *TP* total protein

**Fig. 1:**
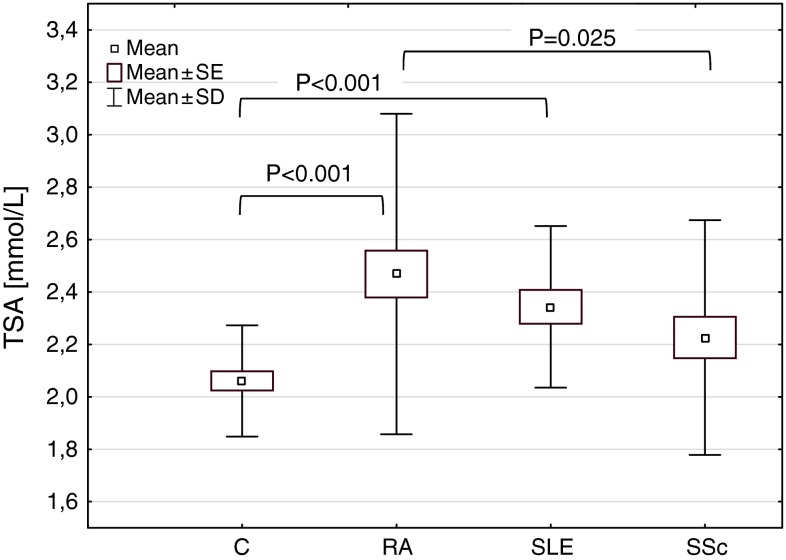
TSA concentrations in patients with rheumatic diseases. *C* controls, *RA* rheumatoid arthritis, *SSc* systemic sclerosis, *SLE* systemic lupus erythematosus

The serum level of TSA in RA (mean ± SD; range 2.47 ± 0.61; 1.69–5.28 mmol/L) and SLE patients (2.34 ± 0.31; 1.87–2.97 mmol/L) was significantly increased compared to controls (2.06 ± 0.21; 1.75–2.54 mmol/L) (*P* < 0.001 for both) (Fig. 1). There was the significant difference in the serum TSA concentration between rheumatic diseases (*H* = 7.199; *P* = 0.027). The mean level of TSA in RA patients was higher than that in SSc patients (2.22 ± 0.45; 1.76–4.30 mmol/L) (*P* = 0.025).

**Fig. 2:**
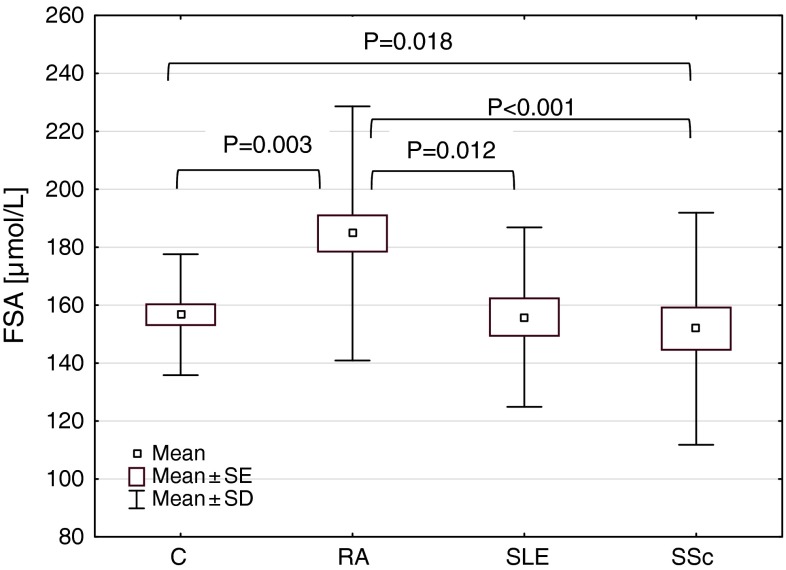
FSA concentrations in patients with rheumatic diseases. *C* controls, *RA* rheumatoid arthritis, *SSc* systemic sclerosis, *SLE* systemic lupus erythematosus

The mean serum level of FSA in RA patients (mean ± SD; range 184.7 ± 43.8; 124.7–331.5 μmol/L) was significantly higher (*P* = 0.003) and in SSc patients (151.9 ± 40.0; 119.0–321.0 μmol/L) significantly lower (*P* = 0.018), than that in the controls (156.7 ± 20.9; 114.9–195.9 μmol/L) (Fig. 2). There were significant differences in the serum FSA concentration between rheumatic diseases (*H* = 20.252; *P* < 0.001). The mean level in RA patients was significantly higher than that in SLE patients (155.9 ± 31.0; 115.8–225.5 μmol/L) (*P* = 0.012) and in SSc patients (*P* < 0.001).

There were positive correlation between positive acute-phase proteins, CRP, AGP, HP, AAT and the levels of TSA and FSA, in RA and SSc patients (Table 3). In SLE patients, there was only the positive correlation HP and AAT with TSA. The negative acute-phase proteins, transferrin, correlated negatively with TSA and FSA only in RA patients. In RA patients, there was the positive correlation of TSA and FSA with DAS 28 (*R* = 0.326 and *R* = 0.538, respectively). The further analysis revealed that for these relations were responsible mainly ESR and VAS. The higher relationship FSA than TSA with DAS 28 comes from the additional correlation of FSA with t28 and s28. RF does not correlate with TSA and FSA in any rheumatic diseases.

**Table 3:** The correlation between acute-phase proteins and TSA and FSA

	TSA			FSA		
	RA	SLE	SSc	RA	SLE	SSc
CRP	*R* = 0.547 *P* < 0.001	*R* = 0.335 *P* = 0.117	*R* = 0.543 *P* = 0.001	*R* = 0.661 *P* < 0.001	*R* = 0.298 *P* = 0.167	*R* = 0.528 *P* = 0.002
AGP	*R* = 0.626 *P* < 0.001	*R* = 0.323 *P* = 0.132	*R* = 0.443 *P* = 0.011	*R* = 0.675 *P* < 0.001	*R* = 0.194 *P* = 0.375	*R* = 0.413 *P* = 0.023
HP	*R* = 0.611 *P* < 0.001	*R* = 0.442 *P* = 0.034	*R* = 0.618 *P* < 0.001	*R* = 0.638 *P* < 0.001	*R* = 0.125 *P* = 0.570	*R* = 0.538 *P* = 0.002
AAT	*R* = 0.507 *P* < 0.001	*R* = 0.567 *P* = 0.004	*R* = 0.530 *P* = 0.002	*R* = 0.507 *P* < 0.001	*R* = 0.371 *P* = 0.081	*R* = 0.577 *P* < 0.001
TRF	*R* = −0.416 *P* = 0.003	*R* = 0.123 *P* = 0.593	*R* = −0.308 *P* = 0.086	*R* = −0.570 *P* < 0.001	*R* = −0.214 *P* = 0.351	*R* = −0.239 *P* = 0.202

Data are Spearman rank correlation coefficients (R) and *P* values. *RA* rheumatoid arthritis, *SLE* systemic lupus erythematosus, *SSc* systemic sclerosis; *P* < 0.05 significant difference; *CRP* C-reactive protein, *AGP* α1-acid glycoprotein, *HP* haptoglobin, *AAT* α1-antitrypsin, *TRF* transferrin

TSA and FSA levels did not differ according to disease activity in SLE patients evaluated by means of PGA scores (*H* = 3.926, *P* = 0.140 for TSA and *H* = 0.256, *P* = 0.879 for FSA).

The sensitivity, specificity, PPV, NPV and the area under the ROC curve (AUC) of TSA and FSA in RA were lower than that of CRP and anti-CCP (Table 4). The diagnostic power (ACC) of TSA and FSA in RA was also lower than that of CRP and anti-CCP. The sensitivity, specificity, PPV and NPV of TSA in SLE were similar to that in RA. The diagnostic power of FSA in SSc was similar to that in RA.

**Table 4:** The diagnostic power of TSA and FSA in RA, SLE and SSc

Test	Sensitivity (%)	Specificity (%)	ACC (%)	PPV (%)	NPV (%)	AUC (±SE)
CRP (RA)	84.8	100	90.5	100	80.0	0.965 ± 0.018
Anti-CCP (RA)	83.7	100	88.4	100	71.4	0.928 ± 0.031
TSA (RA)	66.0	85.3	74.1	86.1	64.4	0.748 ± 0.055
FSA (RA)	61.2	76.5	67.5	78.9	57.8	0.693 ± 0.057
TSA (SLE)	69.6	85.3	78.9	76.2	80.6	0.786 ± 0.063
FSA (SSc)	79.4	63.3	71.9	71.1	73.1	0.673 ± 0.071

*ACC* accuracy, *PPV* positive predictive value, *NPV* negative predictive value, *AUC* area under ROC curve, *SE* standard error

## Discussion

Existing data provide evidence that acute-phase proteins are involved in the inflammation and that effects are time-, concentration- and molecular conformation-dependent, especially are related with the status of glycosylation [14]. From the other hand, the alterations in the glycosylation of plasma proteins may be involved in the etiology of rheumatic diseases [1, 2]. There were a confirmed information about the changes in the different forms of protein glycosylation in rheumatic diseases. These involve galactosylation, fucosylation, mannosylation, sialylation and branching [8, 9, 15–17]. From this reason, human glycoproteins exist in many different glycoforms. The end position on N-glycans is often occupied by sialic acid and is known to be a biochemical marker for the diagnosis of glycosylation abnormalities in several pathological conditions, including rheumatoid diseases. Therefore, we tested the concentration of total sialic acid (TSA), as a non-specific marker of inflammation, and the concentration of free sialic acid (FSA), as a marker of sialylation disturbances in the sera of patients with RA, SLE and SSc.

We denote that the TSA and FSA levels in the sera of patients with RA were elevated in comparison with the controls. The results concerning TSA are in accordance with previously obtained by other authors [18, 19]. Additionally, the level of sialic acid could reflect the progression and the activity of rheumatoid arthritis as indicated in the analysis of correlation of TSA and FSA with the value of DAS 28. The analysis shows that in RA patients, TSA and FSA changed parallel with the alterations of RA activity measured by means of DAS 28. The detailed analysis indicated that mainly ESR and VAS are responsible for these correlations. Other authors suggested that a good correlation between ESR and sialic acid could be a reason for use TSA in place of ESR as a marker of disease activity in RA patients [19]. Increased serum sialic acid has also been reported in inactive RA classified according to ACR criteria for clinical remission of disease [12]. In our study, only two patients have the value of DAS 28 less than 2.6 that implies remission of disease. The other authors indicate on the association of relative sialylation of serum and synovial glycoproteins (e.g., fibronectin) with the progression and activity of rheumatoid arthritis [7]. In contrast to RA activity, the activity of SLE evaluated by PGA scores does not affect the serum levels of TSA and FSA.

This study demonstrates the association between the changes in TSA and FSA concentration and the levels of glycoproteins recommended as acute-phase proteins, especially in RA and SSc patients. The increased concentration of TSA in rheumatic diseases can concern the increased level of sialylated glycoproteins such as α1-acid glycoprotein, haptoglobin and α1-antitrypsin. These are called as positive proteins of acute-phase reaction and can be increased several-fold in response to inflammation, infection, and systemic tissue injury [20]. A good positive correlation between TSA and positive proteins of acute-phase reaction reflects the linear association between the increase in TSA and disease activity. Additionally, the one non-glycosylated protein, CRP, also correlated with TSA and FSA, and this may suggest that both TSA and FSA can be treated as a non-specific markers of inflammation during rheumatic diseases (RA and SSc) [21, 22].

The most widely used indicators of the acute-phase response are the erythrocyte sedimentation rate and the plasma C-reactive protein concentration. ESR is an indirect measurement of plasma acute-phase protein concentrations and can be influenced by the size, shape, and the number of erythrocytes, as well as by other plasma constituents such as immunoglobulins. As has been shown in Table 1, in only SLE patients, IgG level was higher than that in the healthy persons but not differs from that in RA patients. From the other hand, systemic lupus erythematosus is one exception to the generalization that C-reactive protein concentrations correlate with the severity of inflammation [23]. Many patients with active systemic lupus erythematosus do not have high plasma concentrations of CRP but do have marked increases during bacterial infection. Therefore, CRP concentration in SLE patients might be lower than that in RA patients. The cause of low CRP concentrations, incompatible with the inflammatory process, can be due to its binding to anti-CRP antibodies (mostly in class IgG) with the formation of immunological complexes [24]. It can be speculated that the low CRP concentration accompanying active inflammation in SLE patients should be the result the genetic polymorphism of single nucleotide in the promoter region of the C-reactive protein gene [25].

The elevated results of CRP in SSc patients in our study were observed in 34.4 % cases and elevated ESR results in 43.7 % of subjects. Although, the mean CRP and ESR levels were lower than that in RA patients, the frequency of elevation of these parameters in our study is higher than in the patients studied by the Canadian Scleroderma Research Group [26]. The occurrence of elevated CRP results in these studies was 25.7 % and elevated ESR results 38.2 %. As was established in these large cohorts, an elevation of CRP level is more common in the diffuse cutaneous SSc subset, especially with early disease duration (≤3 years). More than half the patients suffered from diffuse cutaneous SSc (18 cases), and equally half the patients had disease lasting shorter than 3 years.

The diagnostic power of TSA and FSA resulting from ROC analysis indicates that these tests have lower sensitivity, specificity, positive and negative predictive values, and the area under the ROC curves than that of CRP and anti-CCP in rheumatic disorders. CRP is generally consider as a marker of inflammation, but Maury et al. [18] investigated that CRP elevation during SLE was not accompanied by recognized intercurrent infection or other superimposed cause of tissue injury and inflammation in 61 % of instances. In those studies, CRP was increased (above 5 mg/L) in 33 % of patients. In our study, there was 42 % (10 cases) of SLE patients with the value of CRP higher than 5 mg/L, than majority of them (7 cases) suffered from renal insufficiency with proteinuria.

From the other hand, the changes in free sialic acid levels can be consider as a result of disturbances in glycosylation of sialylated proteins. Therefore, its correlation with sialylated glycoproteins and additionally with CRP can suggest the FSA reflects the abnormalities in glycosylation of protein caused by activity of inflammation during rheumatic diseases. The negative protein of acute-phase reaction, transferrin, also correlated with TSA and FSA concentration, but there was a negative correlation. This phenomenon was observed only in RA patients. The glycoprotein sensitive to alteration in glycosylation is α1-acid glycoprotein, because contains 42 % carbohydrate in weight, and the glycan component is made by five heteropolysaccharides N-linked to the protein chain [27]. The oligosaccharide moiety expressed on the surface of AGP is extensively heterogeneous in sialylation, fucosylation and branching degree [28]. Therefore, the several glycoforms can be detected in healthy human plasma [29]. Altered glycosylation patterns in plasma AGP and haptoglobin have been found to be associated with the pathogenesis of rheumatoid arthritis [9]. RA patients showed the significant variations in the mannosylation patterns in AGP and HP.

It should be not generalized that the alterations of proteins glycosylation in rheumatic diseases are quite uniform. For example, glycosylation abnormalities of IgG in rheumatic diseases are different [2]. Testing the relationship between exposed galactose and N-acetylglucosamine on IgG from RA, juvenile chronic arthritis (JCA) and SSc patients, the different changes were found. In patients with RA and JCA, the galactose and N-acetylglucosamine are inversely related but in SSc patients, galactose expression on IgG decreased while N-acetylglucosamine remained similar to normal level. The oligosaccharide profile of IgG glycans (proportion of digalactosyl-fucosylated to agalactosyl-fucosylated structures) from RA patients was also significantly different from JCA and SLE patients. In our study, the level of FSA in SSc patients was decreased in contrast to RA patients where FSA level was increased. Also, the TSA level in RA patients was different from SLE and SSc patients. These changes suggest that the unique pattern of glycosylation is associated with different rheumatological changes.

Transferrin, the negative protein of acute-phase response, also alters the glycosylation pattern in rheumatoid arthritis [8]. Although, the concentration of total transferrin diminishes in RA, SLA and SSc patients, the changes in glycosylation can contribute to increased level of sialic acid. In RA patients was observed the significant shift toward the highly branched and sialylated glycan chains of transferrin. These results indicate that the changes in synthetic rate of transferrin as the effect of acute-phase response and the changes in glycosylation are regulated independently.

In conclusion, the changes in the serum TSA and FSA could reflect the acute-phase response (increase of positive acute-phase proteins) and the alterations in glycosylation/sialylation patterns of glycoproteins induced by acute-phase response. The changes in TSA and FSA levels can also reflect the disease activity in RA and SSc.
